# Identification of differentially expressed genes induced by *Bamboo mosaic virus *infection in *Nicotiana benthamiana *by cDNA-amplified fragment length polymorphism

**DOI:** 10.1186/1471-2229-10-286

**Published:** 2010-12-27

**Authors:** Shun-Fang Cheng, Ying-Ping Huang, Zi-Rong Wu, Chung-Chi Hu, Yau-Heiu Hsu, Ching-Hsiu Tsai

**Affiliations:** 1 Graduate Institute of Biotechnology, National Chung Hsing University, Taichung, 40227, Taiwan; 2 Graduate Institute of Medical Laboratory Science and Biotechnology, China Medical University, Taichung, 404, Taiwan

## Abstract

**Background:**

The genes of plants can be up- or down-regulated during viral infection to influence the replication of viruses. Identification of these differentially expressed genes could shed light on the defense systems employed by plants and the mechanisms involved in the adaption of viruses to plant cells. Differential gene expression in *Nicotiana benthamiana *plants in response to infection with *Bamboo mosaic virus *(BaMV) was revealed using cDNA-amplified fragment length polymorphism (AFLP).

**Results:**

Following inoculation with BaMV, *N. benthamiana *displayed differential gene expression in response to the infection. Isolation, cloning, and sequencing analysis using cDNA-AFLP furnished 90 cDNA fragments with eight pairs of selective primers. Fifteen randomly selected genes were used for a combined virus-induced gene silencing (VIGS) knockdown experiment, using BaMV infection to investigate the roles played by these genes during viral infection, specifically addressing the means by which these genes influence the accumulation of BaMV protein. Nine of the 15 genes showed either a positive or a negative influence on the accumulation of BaMV protein. Six knockdown plants showed an increase in the accumulation of BaMV, suggesting that they played a role in the resistance to viral infection, while three plants showed a reduction in coat protein, indicating a positive influence on the accumulation of BaMV in plants. An interesting observation was that eight of the nine plants showing an increase in BaMV coat protein were associated with cell rescue, defense, death, aging, signal transduction, and energy production.

**Conclusions:**

This study reports an efficient and straightforward method for the identification of host genes involved in viral infection. We succeeded in establishing a cDNA-AFLP system to help track changes in gene expression patterns in *N. benthamiana *plants when infected with BaMV. The combination of both DNA-AFLP and VIGS methodologies made it possible to screen a large number of genes and identify those associated with infections of plant viruses. In this report, 9 of the 15 analyzed genes exhibited either a positive or a negative influence on the accumulation of BaMV in *N. benthamiana *plants.

## Background

Most steps involved in plant virus infection, such as the translation of viral genes, the replication of the viral genome and the movement of the viral genome/virion, involve interactions between relatively few viral components and a much more complex pool of host factors [1]. Studies of viral-host interactions provide insight into the life cycle of viruses and could help to devise strategies to tackle viral epidemics among plants. Identification of differentially expressed genes in plants during viral infection can help us to understand the defense systems employed by plants as well as the mechanisms behind the adaption of viruses to plant cells.

Plants are known to defend themselves against attacks from pathogens, such as viruses, bacteria, fungi, invertebrates, and sometimes other plants, by altering the host gene expression [2,3]. Compared to other pathogens, viruses are a particularly serious threat, due to their high mutation rate, which makes them better able to evade host defense systems. Plant viruses use a variety of strategies to promote infection in susceptible hosts. These strategies involve well-documented modifications to host cells such as the formation of replication complexes [4], the suppression of post-transcriptional gene silencing [5], alteration of cell-to-cell trafficking [6-9], and interference with the regulation of host cell cycle [10].

Plant viruses have three prerequisites to survive. First, they must replicate in the initially infected cell. Second, they must move into adjacent cells and the vascular system. Third, they must escape from or suppress the host defense system, by means such as post transcriptional gene silencing [11]. In turn, plants express resistance genes and/or activate systemic acquired resistance to fight the invading viruses [12]. These resistance responses typically involve dramatic changes in the expression of host proteins, such as pathogenesis related (PR) or hypersensitive response (HR) related genes, receptor-like kinases, and serine/threonine kinases [13].

*Bamboo mosaic virus *(BaMV), a single-stranded positive sense RNA virus, is a member of the *potexvirus *genus in the *Flexsiviridae *family. The 6366-nt genome of BaMV comprises a 5'-end m^7^GpppG structure, a 3'-end poly (A) tail, 5'- and 3'-untranslated regions (UTR), and five open reading frames (ORF) [14]. ORF1 encodes a 155-kDa polypeptide with three functional domains, *i.e. *the capping enzyme domain [15-17], an RNA helicase-like domain with RNA 5' triphosphotase and NTPase activities [15,18,19], and an RNA-dependent RNA polymerase domain [20]. ORFs 2, 3 and 4, encoding proteins of 28, 13, and 6 kDa, respectively [14] are required for viral cell-to-cell movement [21,22]. The product of ORF5 is the 25-kDa coat protein. Host factors, such as chloroplast phosphoglycerate kinase, which interacts with the BaMV 3' UTR (identified by UV-crosslinking), may play a positive role in the accumulation of BaMV accumulation in *N. benthamiana *[23]. A putative methyltransferase interacting with RdRp, identified by the yeast two-hybrid system plays a negative role in the accumulation of BaMV [23,24].

This study used cDNA-amplified fragment length polymorphism (AFLP) to identify differentially expressed genes during BaMV infection in *N. benthamiana*. The cDNA-AFLP technique is an efficient, sensitive, and reproducible technology offering several advantages over other PCR methodologies, such as a high degree of selectivity against rare mRNA species [25,26]. The *Tobacco rattle virus *(TRV)-based silencing system was used to knock down the expression of differentially expressed genes obtained by cDNA-AFLP. This study examines and discusses the effects of gene-specific knockdowns on BaMV infection.

## Results

### Screening of BaMV infection-induced genes in N. benthamiana by cDNA-AFLP

Total RNA was extracted from the mock- and BaMV-inoculated leaves 1, 3, 5, and 7 days post inoculation, to identify differentially expressed genes in *N. benthamiana *plants following infection with BaMV. To avoid genomic contamination of the DNA and to enhance the efficiency of reverse transcription, we generated the cDNA from oligo (dT)-purified mRNAs and confirmed the efficiency of synthesizing cDNA on a 5% polyacrylamide gel before proceeding with the production of a standard cDNA-AFLP template [27].

**Figure 1:**
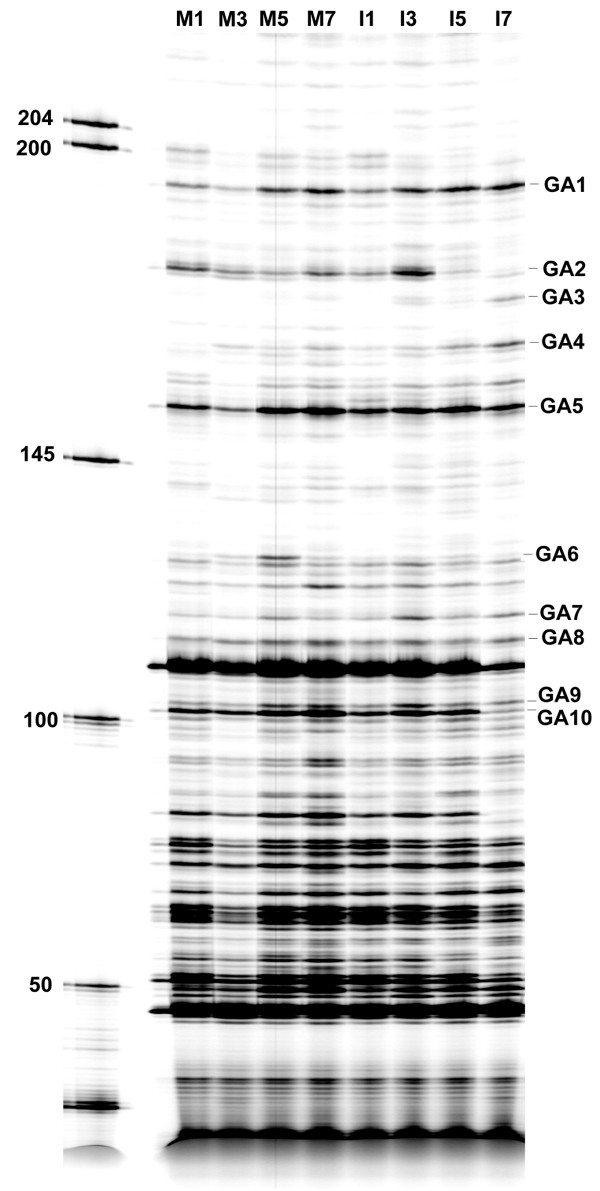
**The cDNA-AFLP profile in BaMV- and mock-transfected *N. benthamiana *leaves**. RNA samples prepared from Mock- (M) and BaMV RNA-inoculated (I) leaves on 1, 3, 5, or 7 dpi were subjected to cDNA-AFLP analysis. Fluorescently labeled cDNA-AFLP fragments generated using the T-CA/M-GA primer pair were separated on a 6.5% polyacrylamide denaturing gel containing 8 M urea and imaged with a fluorescence scanner. The DNA size markers (bp) are indicated on the left side of the gel. The cDNA fragments detected at differential levels and eluted afterwards for further studies were marked with dash lines and designated as GA1 to 10 on the right side of the gel.

To rule out false-positive signals in cDNA-AFLP, we compared the products from two different batches of mRNA, derived from two independent inoculation experiments together on the same gel. In this study, we used eight different primer pairs, T-AC/M-AC, T-AC/M-AG, T-AC/M-CA, T-AC/M-CT, T-AC/M-GA, T-AC/M-GT, T-AC/M-TC, and T-AC/M-TG, to generate the cDNA expression profiles through selective amplification of PCR (Figure 1). Identifying the cDNA fragments of differential levels was simple when lined up together as shown in Figure 1, from which we analyzed the amplified products derived from the T-CA/M-GA primer pair. We assigned positive bands only when the same banding profiles occurred in both batches. The eight primer pairs allowed detection of approximately 90 differentially expressed cDNA bands. Separation of these fluorescently labeled cDNA-AFLP fragments using 6.5% polyacrylamide sequencing gel, imaged with a fluorescent scanner, and eluted from the gel, identified 49 fragments for up-regulation and 41 for down-regulation, following inoculation with BaMV (Table 1).

**Table 1:** A summary of the differentially expressed cDNA fragments isolated with each selective primer pair.

Selective primer	Up regulated	Down regulated
T-AC/M-AC	3	8
T-AC/M-AG	5	7
T-AC/M-CA	11	1
T-AC/M-CT	9	6
T-AC/M-GA	7	3
T-AC/M-GT	8	4
T-AC/M-TC	2	7
T-AC/M-TG	4	5

total	49	41

### Identification of major cDNA species from bands containing multiple genes

We next amplified and cloned the cDNA bands eluted from the cDNA-AFLP gels; DNA sequencing of 6 to 18 clones from each cloning revealed the identity of the cDNA inserts. Sequencing results from approximately 944 clones, indicated that two-thirds (62/90) of the cropped gel fragments contained cDNAs of multiple genes (Additional file 1). These results had been expected, because the gel fragments included any cDNAs in the region. Therefore, further analysis was required to confirm the identity of the genes differentially expressed between mock- and BaMV-inoculated samples.

**Figure 2:**
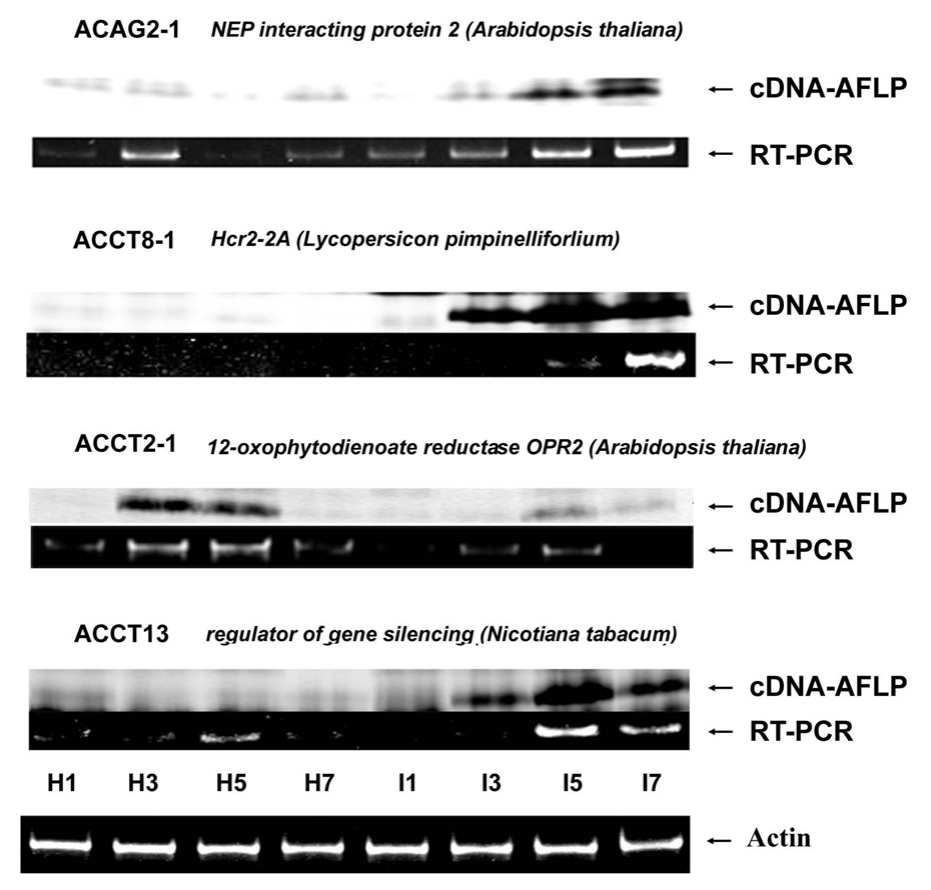
**RT-PCR analysis of the expression profile of the cDNA-AFLP-derived cDNA fragments**. ACAG2-1-, ACCT8-1-, ACCT2-1-, ACCT13-, and actin-specific RT-PCR analysis was carried out using RNA samples prepared from Mock- (M) and BaMV RNA-inoculated (I) leaves on 1, 3, 5, or 7 dpi. The corresponding signal in a cDNA-AFLP analysis is included for each target.

Logically, the clone identified at the highest frequency using DNA sequencing would correspond to the differentially expressed cDNA detected in each gel fragment (Additional file 1). However, there was the possibility of skewed efficiency in the process of cloning the cDNA fragments. Target-specific semi-quantitative RT-PCR was performed to examine whether the expression pattern of the major cDNA species identified in each band, was correlated with the signals in the cDNA-AFLP profile (Figure 2). A third batch of independently inoculated plants provided the mRNA templates used for this experiment. We designed gene-specific primers according to the DNA sequences of the major cDNA clones for more than 10 bands (Additional file 1). Figure 2 shows representative results of RT-PCR analysis including those of ACAG2-1, ACCT8-1, ACCT2-1, and ACCT13. Overall, the expression patterns for all examined targets were consistent with those in the cDNA-AFLP profile. Therefore, we tentatively assigned the major cDNA species identified from each band as representative of cDNA in all 90 bands (Table 2).

**Table 2:** Transcript-derived fragments identified by cDNA-AFLP analysis and differentially expressed between Mock- and *Bamboo mosaic virus*-inoculated *Nicotiana benthamiana* plants.

**TDF**^**a**^	**ID**^**b**^	Length (bp)	**Expression**^**c**^	**Protein candidate**^**d**^	**Ratio**^**e**^	**E value**^**f**^
Function: cell rescue, defense, death, and ageing:						
ACTC1-1	AAM08661.1	207	-	putative disease resistance protein [*Oryza sativa *Japonica]	9/15	
ACTC3-1	AAP03879.1	174	-	Avr9/Cf-9 rapidly elicited protein 216 [*Nicotiana tabacum*]	13/17	1e-05
ACTG3-1	AAB36652.1	213	+	immediate-early salicylate-induced [*Nicotiana tabacum*]	4/7	5e-19
ACCT8-1	AAC78594.1	129	+	Hcr2-2A [*Solanum pimpinellifolium*]	10/12	5e-04
ACCT10	AAA74119.1	121	+	SR1 Nt-rab7b [*Nicotiana tabacum*]	12/12	5e-04
ACAC6	CAA72515.1	205	+	heat shock protein [*Arabidopsis thaliana*]	7/7	1e-21
ACAC8-1	ACG31454.1	144	-	mpv17/PMP22 family protein [*Zea mays*]	5/10	
ACTC8-1	ACG42715.1	120	-	mpv17/PMP22 family protein [*Zea mays*]	12/13	
ACCT13	AAK11255.1	114	+	regulator of gene silencing [*Nicotiana tabacum*]	10/10	5e-04
ACGT10	CAD30209.1	140	+	putative auxin-induced protein 29 [*Arabidopsis thaliana*]	10/10	
ACGT4	AAN63619.1	188	+	thioredoxin h-like protein [*Nicotiana tabacum*]	6/6	4e-09
ACCT14-1	AAC06242.1	102	-	late embryogenis abundant protein 5 [*Nicotiana tabacum*]	5/12	4e-03
ACCT12-1	ABH09088.1	116	-	putative membrane protein [*Artemisia annua*]	6/8	9e-14
ACGT11-1	CAA69901.1	118	+	plasma membrane polypeptide [*Nicotiana tabacum*]	8/10	1e-05
ACGA8-1	AAF24496.1	93	+	FH protein NFH1 [*Nicotiana tabacum*]	6/8	
ACAG2-1	CAC81898.1	218	+	NEP1-interacting protein 2 [*Arabidopsis thaliana*]	9/10	5e-07
ACCA5-1	AAK40224.1	190	+	putative syntaxin of plants 52 [*Oryza sativa *Japonica]	8/10	1e-08
ACCA7-1	ABG73415.1	185	+	chloroplast pigment-binding protein CP29 [*Nicotiana tabacum*]	5/10	4e-05
ACCA8	ABD28323.2	183	+	excinuclease ABC, C subunit[*Medicago truncatula*]	8/8	4e-08
ACCA1-1	AAX95717.1	253	+	protease inhibitor/seed storage/LTP family [*Oryza sativa *Japonica]	5/10	
ACCA10	AAM73656.1	168	+	AER [*Nicotiana tabacum*]	6/6	2e-22
ACAG4-1	AAD32145.1	176	-	Nt-iaa4.5 deduced protein [*Nicotiana tabacum*]	9/10	3e-05
Function: signal transduction:						
ACCT1-1	CAX43672.1	322	-	CDK-activating kinase[*Candida dubliniensis *CD36]	6/9	
ACCT7-1	BAF62637.1	135	+	DELLA protein [*Phaseolus vulgaris*]	10/12	4e-15
ACTG7-1	ABD34616.1	127	-	green ripe-like 1 [*Solanum lycopersicum*]	5/10	5e-04
ACCA2-1	AAS20952.1	204	+	calmodulin binding protein 25 [*Arabidopsis thaliana*]	6/10	5e-06
Function: transcription:						
ACAC2	ABO42262.1	249	-	AT-hook DNA-binding protein [*Gossypium hirsutum*]	8/8	3e-04
ACGA4-1	AAL66977.1	141	+	putative cleavage and polyadenylation specificity factor [*Arabidopsis thaliana*]	8/10	
ACCA6	AAM65499.1	186	+	AP2 domain transcription factor [*Arabidopsis thaliana*]	8/8	2e-05
ACAG11-1	BAC79914.1	57	-	homeobox transcription factor Hox7-like protein [*Oryza sativa *Japonica]	5/10	
ACGA5	AAM14969.1	135	-	putative small nuclear ribonucleoprotein Prp4p [*Arabidopsis thaliana*]	7/7	
ACCT4-1	AAB62807.1	220	+	S-adenosyl-methionine-sterol-C-methyltransferase [*Nicotiana tabacum*]	6/12	2e-34
ACGT1	AAK13103.1	285	-	helicase-like protein [*Oryza sativa *Japonica]	6/6	2e-03
ACGT5-1	AAA35148.1	183	+	transcription factor IIIB [*Saccharomyces cerevisiae*]	6/15	3e-05
Function: metabolism						
ACCT5-1	ACG37370.1	193	-	lysine ketoglutarate reductaselysine trans-splicing [*Zea mays*]	7/12	2e-29
ACTG1-1	ACD13145.1	277	-	TOK1 potassium channel [*Aspergillus fumigatus*]	7/10	
ACAC7-1	AAS46243.1	149	-	xyloglucan endotransglucosylase-hydrolase XTH7 [*Solanum lycopersicum*]	5/9	1e-13
ACCA11-1	ABN09771.1	139	-	glycosyl transferase, family 48 [*Medicago truncatula*]	10/16	1e-13
ACTC4-1	AAA34065.1	169	-	chloroplast carbonic anhydrase [*Nicotiana tabacum*]	10/18	6e-15
ACAG10	AAA34065.1	79	-	chloroplast carbonic anhydrase [*Nicotiana tabacum*]	10/10	1e-07
ACAG8	AAY17071.1	108	-	chloroplast carbonic anhydrase [*Nicotiana benthamiana*]	6/6	5e-21
ACGA10-1	BAA25639.1	76	-	NPCA1 [*Nicotiana paniculata*]	8/10	8e-04
Function: energy						
ACGA9	AAD25541.	84	+	fructose-1,6-bisphosphatase precursor [*Solanum tuberosum*]	8/8	9e-08
ACGT2-1	CAA41713.1	193	-	photosystem II 23 kDa polypeptide [*Nicotiana tabacum*]	5/6	3e-20
ACGT3-1	AAA34053.1	190	+	beta-1,3-glucanase [*Nicotiana tabacum*]	5/6	2e-15
ACGT8-1	CAJ32461.1	160	+	putative chloroplast cysteine synthase 1 [*Nicotiana tabacum*]	10/15	1e-08
ACGT9-1	CAX42612.1	143	+	NADPH-dependent 1-acyl dihydroxyacetone [*Candida dubliniensis *CD36]	7/10	3e-23
ACAG5-1	AAM28014.1	158	+	granule-bound starch synthase [*Peraphyllum ramosissimum*]	7/16	1e-07
ACCA4	BAA28625.1	194	+	aldehyde oxidase [*Arabidopsis thaliana*]	10/10	2e-07
ACAG1	CAA74359.1	269	-	ferredoxin--NADP(+) reductase [*Nicotiana tabacum*]	10/10	7e-38
ACTC2	AAB39547.1	199	-	polygalacturonase isoenzyme 1 beta subunit [*Solanum lycopersicum*]	14/14	1e-28
ACCT2-1	AAC78441.1	295	-	12-oxophytodienoate reductase OPR2 [*Arabidopsis thaliana*]	7/8	2e-32
ACGT12	CAA44267.1	115	-	lipid transferase [*Nicotiana tabacum*]	8/8	6e-10
ACAG9	CAA44267.1	97	-	lipid transferase [*Nicotiana tabacum*]	10/10	7e-08
Function: translation						
ACTC5-1	CAA77372.1	149	-	ribosomal protein L20 [*Nicotiana tabacum*]	6/17	2e-11
ACTG5-1	ABN08437.1	135	+	ribosomal protein L10 [*Medicago truncatula*]	7/10	9e-16
ACGA2-1	CAA77381.1	154	+	ribosomal protein S3 [*Nicotiana tabacum*]	10/17	1e-03
ACGT7-1	CAA77408.1	165	+	ribosomal protein L23 [*Nicotiana tabacum*]	6/10	2e-25
Function: unclassified						
ACTC9-1	BAC98856.1	117	-	hypothetical protein [*Brassica napus*]	5/12	5e-06
ACTG9	AAW82556.	109	-	hypothetical protein [*Phalaenopsis aphrodite *subsp. formosana]	14/14	
ACAC1-1	CAN77388.1	299	-	hypothetical protein [*Vitis vinifera*]	14/15	4e-41
ACAC5-1	CAN79807.1	240	-	unknown protein [*Vitis vinifera*]	8/10	8e-04
ACCT3-1	CAJ32479.1	270	-	hypothetical protein [*Nicotiana tabacum*]	5/10	1e-06
ACCT9-1	AAK20059.1	126	+	hypothetical protein [*Oryza sativa *Japonica]	6/10	
ACGA1-1	ABW98323.1	174	+	hypothetical protein [*Hemiselmis andersenii*]	10/15	
ACCA3	CAA45741.1	200	+	mRNA C-7 [*Nicotiana tabacum*]	8/8	6e-11
ACAG6	BAD46202.1	131	+	hypothetical protein [*Oryza sativa *Japonica]	8/8	
ACCA9-1	AAM91702.1	174	+	unknown protein [*Arabidopsis thaliana*]	4/12	5e-14
No significant match						
ACTG4		148	+	No significant match	8/8	
ACTG6		134	-	No significant match	8/8	
ACTG2-1		271	-	No significant match	5/12	
ACAC3		246	-	No significant match	7/7	
ACAC4-1		245	+	No significant match	4/7	
ACTG8-1		117	+	No significant match	4/12	
ACAC9-1		138	+	No significant match	5/8	
ACAC11		118	-	No significant match	8/8	
ACAC10-1		123	-	No significant match	7/12	
ACCT6-1		151	+	No significant match	10/12	
ACCT11-1		119	+	No significant match	10/12	
ACGA3-1		151	+	No significant match	6/10	
ACCT15-1		96	+	No significant match	6/12	
ACTC6-1		135	+	No significant match	9/18	
ACTC7-1		130	+	No significant match	6/12	
ACGA6-1		107	-	No significant match	7/14	
ACGA7		97	+	No significant match	8/8	
ACGT6		181	-	No significant match	8/8	
ACAG7-1		115	-	No significant match	5/8	
ACAG3-1		186	+	No significant match	7/12	
ACCA12-1		126	+	No significant match	10/14	
ACAG12-1		47	+	No significant match	6/10	

^a^TDF: transcript-derived fragment

^b^ID: accession number of analogues identified by Tblastx

^c^Expression: up-regulated (+) or down-regulated (-) cDNA-AFLP signals detected in virus-infected leaves, compared to mock-infected leaves ^d^Protein candidate: Tblastx hit with the best E value

^e^Ratio: the number of the clone over the number of total clones sequenced

^f^E value: only the value lower than 0.001 (1e-03) were shown according to statistic analysis with extremely significant hit

### Sequence analysis of differentially expressed cDNA fragments

Sequence analysis of the major cDNA species listed in Table 2 revealed that twenty-two of the 90-cDNA fragments shared no significant homology with any known sequences found in the databases. On the other hand, we found analogs for 68 cDNA fragments of which more than two-thirds were sequences derived from *N. tabacum*, Arabidopsis, and rice (Table 2). Among these, 53 led to blast matches of biological significance as suggested by the E-values (Table 2). Table 2 lists the genes categorized according to function: twenty-two genes were involved in cell rescue, defense, death, and aging; 12 in energy; 8 in transcription; 8 in metabolism; 4 in translation; 4 in signal transduction, and 10 could not be classified. Interestingly, three of the genes, namely the mpv17/PMP22 family protein (ACAC8-1 and ACTC8-1), chloroplast carbonic anhydrase (ACAG8, ACAG10, and ACTC4-1), and lipid transferase (ACGT12 and ACAG9) were isolated from different selective primer sets, which remarkably led to identical cDNA-AFLP expression patterns for each target (Table 2). These results implied that the cDNA-AFLP technique is a reliable and reproducible means to identify differentially expressed genes.

### Effect of gene-specific knockdown on the accumulation of BaMV

To investigate the roles of the differentially expressed genes identified by cDNA-AFLP analysis in BaMV infection cycle, the TRV VIGS system [28], which has been used widely to knock down homologous genes in *N. benthamiana *[29], was adopted to generate gene-specific knockdown plants. We evaluated the effects of lowered expression levels of individual host genes on the replication of BaMV, i.e. viral RNA and the accumulation of protein.

**Figure 3:**
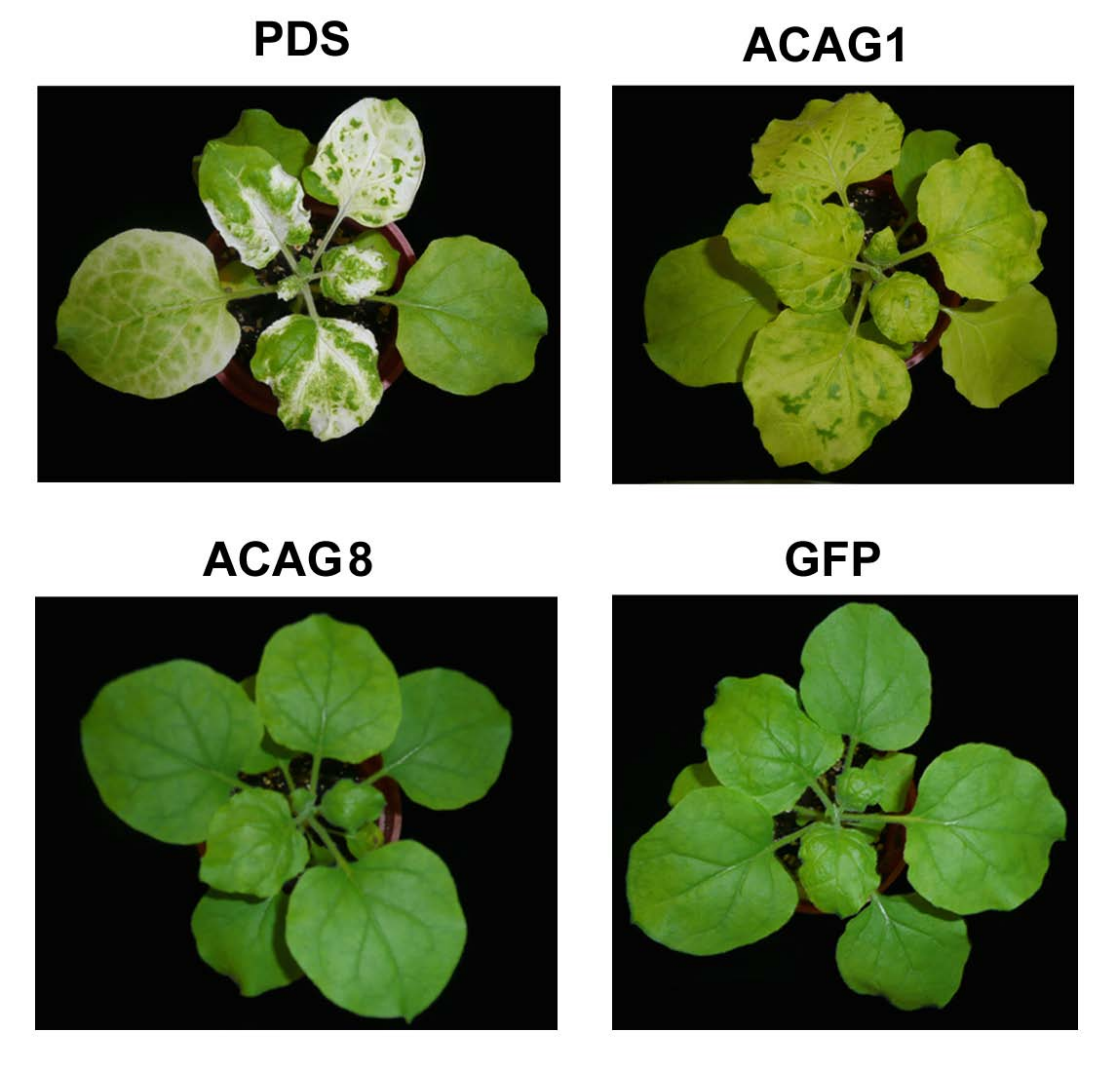
**Phenotypes of gene-specific knockdown plants generated by the TRV VIGS system**. Transcription of ACAG1 and ACAG8 in *N. benthamiana *plant was introduced by the TRV vector to knock down expression of the corresponding host genes. The PDS plant in which *phytoene desaturase *was knocked down served as a positive control. The GFP plant in which the *green fluorescent protein *gene was introduced was included as a negative control.

**Figure 4:**
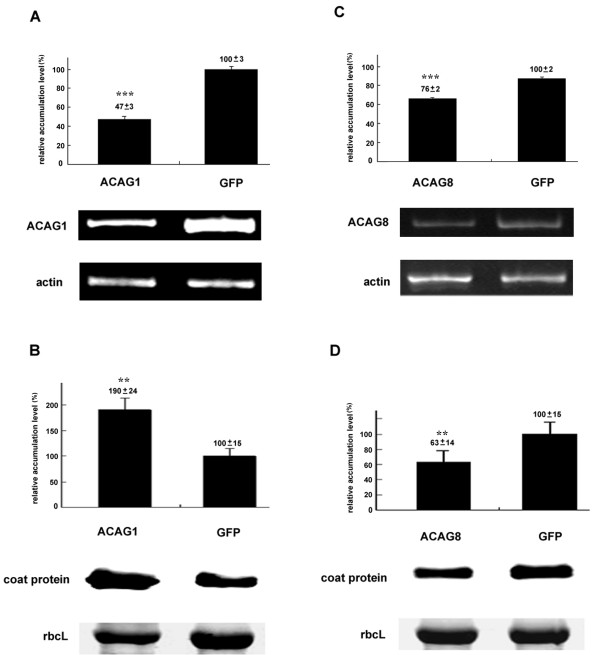
**RT-PCR analysis of host gene expression and Western blotting analysis of BaMV coat protein accumulation in ACAG1- and ACAG8-knockdown plants**. ACAG1- and ACAG8-knockdown plants were inoculated with viral RNA. The GFP plant was included as the negative control. RNA and protein extracts of leaves inoculated with viral RNA were harvested on 5 dpi. The RNA extracts were subjected to ACAG1- (A) or ACAG8-specific (C) semi-quantitative RT-PCR. RT-PCR data was normalized to the levels of actin. Protein extracts were analyzed for BaMV coat protein accumulation by Western blotting (B and D). The Rubisco large subunit (rbcL) was included as the loading control for normalization. For all experiments, the levels detected in the GFP control plants on 5 dpi were set as 100%. Representative results are shown under the statistical results showing the average of the relative levels of AGAC1 mRNA (A), ACAG8 mRNA (C) and, BaMV coat protein (B and D) with standard deviations derived from at least three independent experiments. Asterisks indicate statistically significant differences compared with the control indicated as GFP (**p < 0.05, ***p < 0.001).

**Figure 5:**
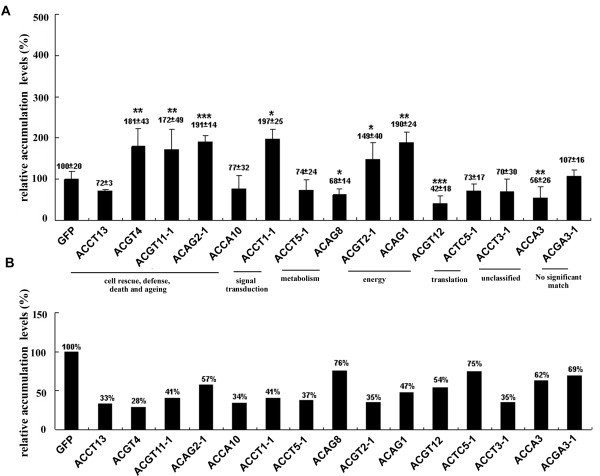
**BaMV coat protein accumulation and the knockdown efficiency in each gene knockdown plants**. The specific gene knockdown plants indicated below each statistic bar were inoculated with BaMV RNA. Protein and RNA extracts were analyzed for coat protein accumulation by Western blotting (A) and specific gene knockdown efficiency by semi-quantitative RT-PCR (B), respectively. The relative coat protein or RNA accumulation levels compared to that of control plants indicated as GFP (100%) were analyzed. The accumulation levels with standard deviations and their significances (*t*-test) of coat protein accumulation were shown above each statistic bar. Asterisks indicate statistically significant differences compared with the control indicated as GFP (*p < 0.01, **p < 0.05, ***p < 0.001).

To assess the effect of the TRV vector in *N. benthamiana*, GFP or Luciferase ORF (non plant-derived DNA) were introduced to the pTRV2 vector to serve as a control. Fifteen genes, picked randomly from each assigned functional category (Table 2) for knockdown experiments showed no significant effect on plant growth or development. Most of these knockdown plants (Table 3) exhibited no difference in morphology to that of the control plants (Figures 3 and Additional file 2). However, yellowing mosaics occurred on leaves of the ACAG1 (a putative ferredoxin-NADP^+ ^reductase; *FNR*) of the knockdown plants (Figure 3). The results of the studies of BaMV regarding ACAG1 and ACAG8 (a putative chloroplast carbonic anhydrase, *cCA*) in the knockdown plants are described here to represent our observations of these 15 knockdown plants (Figures 3 and 4). We used semi-quantitative RT-PCR to assess the knockdown efficiency of the VIGS system (Figures 5 and Additional file 2) and Western blot analysis to determine the accumulation of BaMV coat protein in the inoculated leaves. Results indicate that mRNA levels of the *FNR *gene were reduced to 47% that of the control plants (Figure 4A). Western blot analysis of BaMV coat protein detected a nearly two-fold accumulation in 5 dpi samples in these plants (Figure 4B). The mRNA levels of the *cCA *gene were reduced to 76% (significance in *t*-test) in the knockdown plants (Figure 4C) leading to a reduction in the accumulation of coat protein to 63% that of the control plants (Figure 4D). These results suggest that *FNR *might play a role preventing the accumulation of BaMV, whereas *cCA *could facilitate the accumulation of BaMV.

**Table 3:** BaMV coat protein accumulation in TRV-driven gene-silenced Nicotiana benthamiana plants.

**TDF**^**a**^	**Expression**^**b**^	Protein candidate	**CP**^**c**^	**Significance**^**d**^
ACCT13	+	regulator of gene silencing [*N. tabacum*]	72 ± 3	
ACGT4	+	thioredoxin h-like protein [*N. tabacum*]	181 ± 43	**
ACGT11-1	+	plasma membrane polypeptide [*N. tabacum*]	172 ± 49	**
ACAG2-1	+	NEP1-interacting protein 2 [*Arabidopsis thaliana*]	191 ± 14	***
ACCA10	+	AER [*N. tabacum*]	77 ± 32	
ACCT1-1	-	CDK-activating kinase[*Candida dubliniensis *CD36]	197 ± 25	**
ACCT5-1	-	lysine ketoglutarate reductaselysine trans-splicing [*Zea mays*]	74 ± 24	
ACAG8	-	chloroplast carbonic anhydrase [*N. benthamiana*]	68 ± 14	*
ACGT2-1	-	photosystem II 23 kDa polypeptide [*N. tabacum*]	149 ± 40	**
ACAG1	-	ferredoxin--NADP(+) reductase [*N. tabacum*]	190 ± 24	**
ACGT12	-	lipid transferase [*N. tabacum*]	42 ± 18	***
ACTC5-1	-	ribosomal protein L20 [*N. tabacum*]	73 ± 17	
ACCT3-1	-	hypothetical protein [*N. tabacum*]	70 ± 30	
ACCA3	+	mRNA C-7 [*N. tabacum*]	56 ± 26	**
ACGA3-1	+	No significant match	107 ± 16	

^a^TDF: transcript-derived fragment

^b^Expression: up-regulated (+) or down-regulated (-) cDNA-AFLP signals detected in virus-infected leaves, compared to mock-infected leaves

^c^CP: the coat protein accumulation levels of BaMV in knockdown plants

^d^Significance: Asterisks indicate statistically significant differences compared with the control plants (*p < 0.05, **p < 0.01, ***p < 0.001)

Among the 15 genes analyzed (by VIGS knockdown experiments), we found that the levels of accumulated coat protein significantly increased in six knockdown plants suggesting that these six genes play a role in counteracting BaMV infection (Figures 5A and Table 3). Three knockdown plants showed a significant reduction in the level of coat protein, implying that these three genes play a positive role in the accumulation of BaMV in plants. No statistically significant difference was shown between the six remaining knockdown plants and the control plants when inoculated with BaMV. Interestingly, all of the genes playing a potentially negative role in the accumulation of BaMV within the categories related to cell rescue, defense, death, ageing, signal transduction, and energy. These results suggest that the proteins involved in signal transduction pathways related to pathogen defense might be involved in resistance to BaMV in *N. benthamiana *plants. Finally, approximately three-fifths of the 15 randomly picked, differentially expressed genes showed either a positive or a negative influence on the accumulation of BaMV in plants.

## Discussion

The *Arabidopsis *genetic system is a common choice for the identification of plant genes involved in the interactions with plant-pathogens. Lately, other genomic-scale methods, such as cDNA-AFLP, serial analysis of gene expression, cDNA microarray, and proteomics have been developed to study the interactions associated with plant-pathogens [30]. Among these, cDNA-AFLP is useful in detecting differentially expressed genes when genome sequence or microarray data is unavailable [25]. This method employs two restriction enzymes to generate short fragments in the analysis of AFLP. The choice of restriction enzyme depends on the complexity of the target templates [25]. Commonly, cleavage in cDNA templates involves the use of four-base cutter enzymes to generate fragments of ideal sizes (0.1-1.0 kb). Because of the relatively low complexity of the cDNA, two selective bases for each primer enabled 256 possible primer combinations [25]. With commercially available resources and a few modifications, a high-throughput gene expression detection system can be easily established [13].

In this study, we used eight pairs of primers in the cDNA-AFLP analysis, to isolate 90 differentially expressed genes in BaMV-inoculated plants. However, one of the major drawbacks of this technique is that each banding from the AFLP reaction could comprise more than one cDNA fragment. Therefore, it is important to confirm differential expression of the targets identified by cDNA-AFLP, using techniques such as real time RT-PCR, semi-quantitative RT-PCR, or Northern blotting. Three different batches of mRNAs (two for cDNA-AFLP and the third for confirmation) were extracted from *N. benthamiana *plants prepared independently to reduce the risk of false positive results. Another drawback was that the sequencing of the genome of *N. benthamiana*, the experimental plant used in this study, is not yet complete. Many cDNA fragments of *N. benthamiana *identified by the cDNA-AFLP analysis had no significant match in the database. Although a work around approach is available, in which Arabidopsis or rice is used as the host plant, *N. bentamiana *is still a more suitable organism for the study of BaMV life cycle. Furthermore, host gene expressions in *N. benthamiana *can be knocked down by the VIGS systems [28,29,31] to determine whether these differentially expressed genes have any influence on the accumulation of BaMV.

Knocking down the expression levels of these host genes using the VIGS system enables the identification of the novel functions of genes in pathogen-host interactions. *FNR *is down regulated upon infection with BaMV (Table 2). The leaves of *FRN *knockdown plants display discoloration similar to that induced by BaMV infection in control plants (Figure 3), suggesting that *FNR *may be involved in the development of viral symptoms. *FNR *may also be a gene associated with innate plant immunity capable of suppressing the accumulation of BaMV, as suggested by the observation that *FNR *knockdown plants shows elevated levels of viral products compared to infected control plants. On the other hand, the expression of the cCA, which catalyzes reversible hydration of CO_2 _in plants, down regulates in response to infection with BaMV. Future investigations could test the hypothesis that *FNR *is a gene associated with innate immunity by studying plants that transiently or permanently over-express *FNR*, to evaluate the effects of higher levels of host *FNR *on the replication of BaMV. Lower levels of accumulated BaMV were detected in *cCA *knockdown plants (Figure 4), suggesting that cCA could be recruited by BaMV to facilitate viral replication. Examination of the effects of *cCA *over-expression on BaMV replication in plants would test this hypothesis.

ACAG2 is another potential pathogen defense gene enhancing the accumulation of BaMV coat protein approximately 2 folds, when knocked down in *N. benthamiana *plants. We have predicted that ACAG2 is a nuclear-encoded polymerase (NEP) interacting-protein (NIP) containing three transmembrane domains and one RING-H2 domain. The RING domain is reported to interact with E3-ubiquitin ligases mediating ubiquitination and degradation of the protein by the proteasome [32].

## Conclusions

The VIGS system helps to identify the roles of differentially expressed genes associated with BaMV infection. We have succeeded in establishing a cDNA-AFLP system to help track the changes involved in gene expression patterns in *N. benthamiana *plants during viral infection. In total, 90 differentially expressed genes were uncovered using eight primer pairs in the analysis in BaMV-infected *N. benthamiana*. Combining both cDNA-AFLP and VIGS methodologies, makes the screening of large numbers of genes possible, to identify those playing a critical role in plant virus infection. In this report, 9 of the 15 genes analyzed exhibited either a positive or a negative influence on the accumulation of BaMV in *N. benthamiana *plants.

## Methods

### Plant material and viral inoculation

Plants (*Nicotiana benthamiana*) were grown in a growth chamber with a 16 h day length at 28°C. Six-week-old plants were mechanically inoculated with 500 ng of BaMV on each leaf. Virus- and mock-inoculated leaves were harvested on day 1, 3, 5 or 7 post-inoculation (dpi).

### Plant mRNAs isolation

Total RNA was extracted from 3 g of leaves. The leaves were ground to powder with liquid nitrogen and mixed with 6 ml of STE buffer (100 mM Tris-HCl, pH 8.0, 100 mM NaCl and 10 mM EDTA), 660 μl of 10% SDS and 180 μl of 100 mg/ml bentonite. The mixture was centrifuged at 12000 rpm for 10 min at 4°C (Sigma model 3MK centrifuge) after three times of phenol/chloroform extraction. Total RNA in the supernatant was ethanol precipitated, stored at -80°C, and subjected to poly(A) RNA isolation by using oligo(dT)-coupled paramagnetic beads. Briefly, 100 μl of the total RNA (75 μg) were heated at 65°C for 2 min to disrupt secondary structure and then placed on ice. About 200 μl of Dynabeads Oligo (dT)25 (Dynal A.S., Oslo, Norway) were washed twice with 100 μl of binding buffer (20 mM Tris-HCl pH 7.5, 1.0 M LiCl, 2 mM EDTA) and resuspended in 100 μl of binding buffer. The beads were incubated with total RNA for 3-5 min at room temperature, washed twice with 200 μl of washing buffer (10 mM Tris-HCl pH 7.5, 0.15 M LiCl, 1 mM EDTA) and resuspend in 10 μl of deionized water to elute the mRNA.

### cDNA synthesis

For the first-strand cDNA synthesis, the 20-μl reaction containing 750 ng of mRNA, 30 pmole of Oligo (dT)_40_, 50 mM Tris-HCl pH 8.3, 75 mM KCl, 3 mM MgCl_2_, 1 mM dNTP,10 mM DTT, and 1 μl of 200 U/μl SuperScript^® ^III Reverse Transcriptase (Invitrogen, Carlsbad, CA, USA)was incubate at 42°C for 90 min. Following removal of the mRNA by alkaline lysis, the cDNA was ethanol precipitated, washed, dried, and dissolved in 10 μl of deionized water. The second-strand cDNA was synthesized in a 10-μl reaction containing 4 μl of first-strand cDNA, 10 mM Tris-HCl (pH 7.5), 5 mM MgCl_2_, 7.5 mM DTT, 10 mM dNTP, and 2.5 units of Klenow polymerase (New England Biolabs, Beverly, MA, USA) at 25°C for 30 min. The enzyme was inactivated with 100 mM EDTA for 20 min at 75°C.

### cDNA-AFLP

cDNA-AFLP was carried out using the AFLP^® ^Expression Analysis Kits (LI-COR Biosciences, Lincoln, NE, USA), according to the protocols provided by the manufacturer. Double-strand cDNA was sequentially digested with *Taq*I at 65°C for 2 hours and with *Mse*I at 37°C for another 2 hours. After inactivation of the restriction enzymes at 80°C for 20 min, 9 μl of the adapter mixture containing the adapters (*Taq*I adapters: 5'GCGCGCCGTAGACTGCGTAC 3', 5'CGGTACGCAGTCTACGGCGCGC3', *Mse*I adapters: 5'GGCCGCCGATGAGTCCTGAG3', 5'TACTCAGGACTCATCGGCGGCC3'), 0.4 mM ATP, 10 mM Tris-HCl pH 7.5, 10 mM Mg(OAc)_2_, 50 mM KOAc, and 6 Weiss units of T4 DNA ligase (New England Biolabs) were added to the restriction digestion mixture and incubated at 20°C for 2 hours.

Subsequently, twenty cycles of pre-amplification were carried out in a 20-μl reaction containing 2.5 μl of 30-fold diluted cDNA template, 100 pmole each of *Taq*I primer (5'GTAGACTGCGTAC3') and *Mse*I primer (5'GATGAGTCCTGAG3'), 0.25 mM dNTP, 1.5 mM MgCl_2_, and 5 units of *Taq *DNA polymerase (Promega, Madison, WI, USA). The PCR thermal cycling consisting of 20 cycles of 94°C for 30 sec, 56°C for 1 min, and 72°C for 1 min was performed on a GeneAmp PCR system 9600 instrument (Applied Biosystems, Foster city, CA, USA). The amplification products (i.e. the secondary template) were diluted 300 folds and subjected to selective amplification. The reaction contained 6 μl of *Taq *DNA polymerase working mix (20 mM Tris-HCl pH 8.4, 1.5 mM MgCl_2_, 100 mM KCl and 0.75 unit *Taq *DNA polymerase), 2 μl of the secondary template, 2 μl of *Mse*I primer, and 0.5 μl of IRDye™ 700-labeled *Taq*I primer. The amplification conditions are as follows: 13 cycles of 94°C for 30 sec, 65°C for 30 sec (temperature increment reduction of 0.7°C per cycle), and 72°C for 1 min, followed by 23 cycles of 94°C for 30 sec, 56°C for 30 sec, and 72°C for 1 min. Samples were denatured at 95°C for 5 min after the addition of stop solution (10 mM NaOH, 95% formamide, 0.05% bromophenol blue, 0.05% xylene cyanol) and separated on a 6.5% KB^Plus™ ^gel. Labeled DNA fragments were visualized and recorded by the automatic DNA Sequencer LI-COR 4300 (LI-COR Biosciences)

### Isolating and sequencing the differentially expressed cDNA fragments

The bands of interest, namely the transcript-derived fragments TDF, were marked on the Odyssey™ Scanner (LI-COR Biosciences), cut out with a sterile razor blade, and soaked in 10 μl of TE buffer (10 mM Tris-HCl pH8.0, 1 mM EDTA). Following a series of freeze-thaw steps, the cDNA fragments were leached out from the gel by centrifugation at the top speed of a microfuge for 20 min at 4°C. Re-amplification of the cDNA fragments was carried out under the same conditions of the pre-amplification step. The PCR products were separated on a 5% polyacrylamide gel and cloned into pGEM^®^-T Easy vector (Promega). DNA sequencing was conducted using the Simultaneous Bi-directional Sequencing (SBS™) method (LI-COR) on a Global IR^2 ^System (LI-COR). DNA sequence homology search within the GenBank^® ^database was performed using BLAST [33].

### Semi-quantitative RT-PCR

First-strand cDNA of RNA prepared from mock- or BaMV-inoculated *N. benthamiana *plants was synthesized with d(T)_39 _primer using SuperScrpt^® ^III reverse transcriptase (Invitrogen, Carlsbad, CA, USA). Four sets of primers were used to confirm the expression profiles of four cDNA fragments identified by cDNA-AFLP, namely ACAG2-1, ACCT8-1, ACCT2-1, and ACCT13. The forward primers are (5'GAACAAAAAAATGGAGTTTTA3'), (5'CGAACTCCCAACTGGCTTTC3'), (5'CTCTGGAAAGGAGAGCAATGTC3'), and (5'GAACGCTTTGATGAGAATAGAGA3') and the reverse primers (5'GTCATTGCTCCTAATAAGGT3'), (5'CTCCTCCAGAAGCAAATAGTTTC3'), (5'CGAACAAATTGGTGTATCC3'), and (5'CTAACTCAACCGCAGCCTTT3'), respectively. PCR amplifications were performed using *Taq *DNA polymerase (Promega) with 28 cycles of 94°C for 30 sec, 55°C for 30 sec, and 72°C for 30 sec. PCR products were separated on a 5% polyacrylamide gel and visualized by EtBr staining.

Primer pairs for ACAG1 (forward, 5'GAGAAAATGAAGGAGAAGGCCC3'; reverse, 5'GCTCTGCCTTCTTCAATTGCTTCTT3') and ACAG8 (forward, 5'GAAGGAAGCTGTGAATGTGTCA3'; reverse, 5'TGGTTAAGTTCATACGGAAAGA3') were used to determine the knockdown efficiency of host genes by VIGS (virus-induced gene silencing). The actin primer pair (forward, 5'GTGGTTTCATGAATGCCAGCA3'; reverse 5'GATGAAGATACTCACAGAAAGA3') was used for normalization of RT-PCR data.

### Virus-induced gene silencing (VIGS)

Two transcript-derived fragments (ACAG1 and ACAG8) were first cloned into pGEM-T Easy vector (Promega) and subcloned into the *EcoR*I site of the pTRV2 vector [28]. The control plasmid pTRV2/mGFP was obtained by subcloning the *Kpn*I-*Xho*I fragment containing the polyhistidine-tagged mGFP5-coding sequence [34] from pBI-mGFP1 into pTRV2. The pTRV2/ACAG1, pTRV2/ACAG8 and pTRV2/mGFP constructs were transformed into *Agrobacterium tumefaciens *C58C1 strain by electroporation.

For agroinfiltration, the *A. tumefaciens *C58C1 containing pTRV1, pTRV2/mGFP, pTRV2/ACAG1, or pTRV2/ACAG8 was cultured to OD_600 _= 1 at 30°C and subjected to induction in 150 μM acetosyringone and 10 mM MgCl_2 _for 2 h at room temperature. Subsequently, the pTRV2/mGFP-, pTRV2/ACAG1- or pTRV2/ACAG8-containing *A. tumefaciens *C58C1 was mixed with the pTRV1-containing *A. tumefaciens *C58C1 at a 1:1 (v:v) ratio. The 2^nd ^and 3^rd ^true leaves were infiltrated with the mixture at the four-leaf stage (seedlings with two cotyledons and two leaves). BaMV virion RNA (1 μg) was inoculated onto the 7^th ^leaf when the plants were mature. Total RNAs and proteins were extracted from the leaves on 5 dpi for subsequent studies.

### Protein detection

Total proteins of the leaves were extracted in 1x Laemmli buffer (2.5 mM Tris-HCl, pH 8.3, 250 mM glycine and 0.1% SDS) and incubated in boiling water for 5 min. Proteins separated by SDS-PAGE were subjected to Western blotting analysis using the polyclonal rabbit anti-BaMV coat protein antibody. The relative levels of the Rubisco large subunit (rbcL) in gels stained with Coomassie Blue were determined and used for the normalization of the Western blotting signals.

## Authors' contributions

SFC prepared cDNAs for AFLP analysis, cloned the isolated cDNA fragments, conducted the VIGS experiments, YPH performed the cloning and sequencing of the cDNAs derived from cDNA-AFLP, ZRW conducted inoculation and harvesting of N. benthamiana plants with BaMV, CCH undertook bioinformatics analysis of sequence data and participated in the discussion, YHH and CHT were project supervisors, participated in the discussion of all experiments of the project and preparation of the manuscript.

All authors read and approved the final manuscript.

## Supplementary Material

Additional file 1 **Table S1: Transcript-derived fragments identified by cDNA-AFLP analysis and differentially expressed between Mock- and Bamboo mosaic virus-inoculated Nicotiana benthamiana plants**. Table S2: The primer set and their sequence for RT-PCR to examine the knockdown efficiency. Click here for file

Additional file 2 **Figure S1 - Phenotypes of gene-specific knockdown plants generated by the TRV VIGS system**. Transcription of ACCA3, ACGT12, or ACAG8 in N. benthamiana plant was introduced by the TRV vector to knock down expression of the corresponding host genes. The Luc plant in which the luciferase gene was introduced was included as a negative control. Figure S2 - RT-PCR analysis of host gene expression in knockdown plants. The knockdown plants as indicated above each lane were inoculated with viral RNA. The GFP plant was included as the negative control. The RNA extracts derived from the leaves inoculated with viral RNA were harvested on 5 dpi and subjected to specific primers indicated on the left for semi-quantitative RT-PCR. RT-PCR data was normalized to the levels of actin. Click here for file
