# Supplementary material for: Identification of differentially expressed genes induced by Bamboo mosaic virus infection in Nicotiana benthamiana by cDNA-amplified fragment length polymorphism
Source: BMC Plant Biol. 2010 Dec 27;10:286. doi: 10.1186/1471-2229-10-286 (PMC3024324; doi:10.1186/1471-2229-10-286)
Supplement: Additional file 1 — Table S1: Transcript-derived fragments identified by cDNA-AFLP analysis and differentially expressed between Mock- and Bamboo mosaic virus-inoculated Nicotiana benthamiana plants. Table S2: The primer set and their sequence for RT-PCR to examine the knockdown efficiency. [file 1471-2229-10-286-S1.PDF]

Table S1. Transcript-derived fragments identified by cDNA-AFLP analysis and differentially expressed between Mock- and *Bamboo mosaic virus*-inoculated *Nicotiana benthamiana* plants.

TDF[a]	ID[b]	Length	Expression[c]	Protein candidate[d]	Ratio[e]	E value[f]
ACTC1-1	AAM08661.1	207	—	putative disease resistance protein [*Oryza sativa* Japonica]	9/15	
-2	BAD82750.1	206		putative terminal ear1 [*Oryza sativa* Japonica]	4/15	
-3		207		no significant match	1/15	
-4		206		no significant match	1/15	
ACTC2	AAB39547.1	199	—	polygalacturonase isoenzyme 1 beta subunit [*Solanum lycopersicum*]	14/14	1e-28
ACTC3-1	AAP03879.1	174	—	Avr9/Cf-9 rapidly elicited protein 216 [*Nicotiana tabacum*]	13/17	1e-05
-2	AAW22873.1	177		putative polyprotein [*Solanum lycopersicum*]	2/17	
-3		178		no significant match	1/17	
-4	ABJ17153.1	176		At1g13710 [*Arabidopsis thaliana*]	1/17	
ACTC4-1	AAA34065.1	169	—	chloroplast carbonic anhydrase [*Nicotiana tabacum*]	10/18	6e-15
-2	CAN78644.1	166		hypothetical protein [*Vitis vinifera*]	8/18	4e-23
ACTC5-1	CAA77372.1	149	—	ribosomal protein L20 [Nicotiana tabacum]	6/17	2e-11
-2		154		No significant match	5/17	
-3		151		No significant match	3/17	
-4	CAN74970.1	153		hypothetical protein [Vitis vinifera]	1/17	7e-11
-5		152		No significant match	1/17	
-6	AAU03364.1	150		chloroplast-specific ribosomal protein [Solanum lycopersicum]	1/17	
ACTC6-1		135	+	no significant match	9/18	
-2		134		no significant match	3/18	
-3		134		no significant match	3/18	
-5		135		no significant match	1/18	

-6		135		no significant match	1/18	
-7	CAN65888.1	133		hypothetical protein [*Vitis vinifera*]	1/18	4e-07
ACTC7-1		130	+	no significant match	6/12	
-2		129		no significant match	3/12	
-3	BAD05466.1	132		unknown protein [*Oryza sativa* Japonica]	2/12	
-4	AAF66136.1	131		unknown protein; 23105-20540 [*Arabidopsis thaliana*]	1/12	
ACTC8-1	ACG42715.1	120	−	mpv17 / PMP22 family protein[*Zea mays*]	12/13	
-2		119		no significant match	1/13	
ACTC9-1	BAC98856.1	117	−	hypothetical protein	5/12	5e-06
-2	ABD96949.1	116		hypothetical protein [*Cleome spinosa*]	3/12	
-3	ACG30390.1	115		hypothetical protein [*Zea mays*]	3/12	
-4		113		no significant match	1/12	
ACTG1-1	CAK40338.1	277	−	contig An09c0140 [*Aspergillus niger*]	7/10	
-2		277		no significant match	1/10	
-3	BAC99804.1	276		putative methylcrotonyl-CoA carboxylase beta [*Oryza sativa* Japonica]	1/10	
-4	AAW41604.1	275		putative multidrug resistance protein fnx1 [*Cryptococcus neoformans* var. neoformans JEC21]	1/10	
ACTG2-1		271	−	no significant match	5/12	
-2	AAN15627.1	271		nucleotide sugar epimerase-like protein [*Arabidopsis thaliana*]	4/12	8e-09
-3		269		no significant match	3/12	
ACTG3-1	AAB36652.1	213	+	immediate-early salicylate-induced[*Nicotiana tabacum*]	4/7	5e-19
-2		214		no significant match	3/7	
ACTG4		148	+	no significant match	8/8	
ACTG5-1	ABN08437.1	135	+	ribosomal protein L10 [*Medicago truncatula*]	7/10	9e-16

-2	AAO26214.1	136		type 5 protein serine/threonine phosphatase 55 [*Solanum lycopersicum*]	3/10	
ACTG6		134	−	no significant match	8/8	
ACTG7-1	ACJ84592.1	127	−	unknown protein [*Medicago truncatula*]	5/10	5e-04
-2	ABK93312.1	124		unknown protein [*Medicago truncatula*]	3/10	8e-04
-3		125		no significant match	2/10	
ACTG8-1		117	+	no significant match	4/12	
-2	AAL32300.1	118		lethal leaf spot 1-like protein [*Solanum lycopersicum*]	3/12	
-3		116		no significant match	3/12	
-4		115		no significant match	2/12	
ACTG9	AAW82556.	109	−	hypothetical protein [*Phalaenopsis aphrodite* subsp. formosana]	14/14	
ACAC1-1	CAN77388.1	299	−	hypothetical protein [*Vitis vinifera*]	14/15	4e-41
-2		297		no significant match	1/15	
ACAC2	ABO42262.1	249	−	AT-hook DNA-binding protein [*Gossypium hirsutum*]	8/8	3e-04
ACAC3		246	−	no significant match	7/7	
ACAC4-1		245	+	no significant match	4/7	
-2	CAN67243.1	244		hypothetical protein [*Vitis vinifera*]	3/7	6e-04
ACAC5-1	CAN79807.1	240	−	unknown protein [*Vitis vinifera*]	8/10	8e-04
-2	BAF01244.1	241		hypothetical protein [*Arabidopsis thaliana*]	1/10	8e-04
-3	ABD32385.2	236		GNS1/SUR4 membrane protein [*Medicago truncatula*]	1/10	4e-29
ACAC6	CAA72515.1	205	+	heat shock protein [*Arabidopsis thaliana*]	7/7	1e-21
ACAC7-1	AAS46243.1	149	−	xyloglucan endotransglucosylase-hydrolase XTH7 [*Solanum lycopersicum*]	5/9	1e-13
-2	CAN73150.1	149		hypothetical protein [*Vitis vinifera*]	2/9	3e-10
-3		150		no significant match	2/9	

ACAC8-1	ACG31454.1	144	−	mpv17 / PMP22 family protein [*Zea mays*]	5/10	
-2	CAB78969.1	146		putative protein [*Arabidopsis thaliana*]	3/10	
-3		145		no significant match	2/10	
ACAC9-1		138	+	no significant match	5/8	
-2	ABI49441.1	136		At1g75240 [*Arabidopsis thaliana*]	3/8	3e-05
ACAC10-1		123	−	no significant match	7/12	
-2	ABO95693.1	124		predicted protein [*Ostreococcus lucimarinus* CCE9901]	4/12	
-3	ACG40144.1	125		hypothetical protein [*Zea mays*]	1/12	
ACAC11		118	−	no significant match	8/8	
ACCT1-1	CAX43672.1	322	−	CDK-activating kinase[*Candida dubliniensis* CD36]	6/9	
-2	AAM51275.1	326		unknown protein [*Arabidopsis thaliana*]	3/9	5e-21
ACCT2-1	AAC78441.1	295	−	12-oxophytodienoate reductase OPR2 [Arabidopsis thaliana]	7/8	2e-32
-2		300		no significant match	1/8	
ACCT3-1	CAJ32479.1	270	−	hypothetical protein [*Nicotiana tabacum*]	5/10	1e-06
-2	AAW82556.1	275		hypothetical protein [*Phalaenopsis aphrodite* subsp. formosana]	4/10	2e-10
-3		273		no significant match	1/10	
ACCT4-1	AAB62807.1	220	+	S-adenosyl-methionine-sterol-C-methyltransferase [*Nicotiana tabacum*]	6/12	2e-34
-2		222		no significant match	2/12	
-3	AAQ04833.1	219		lecithine cholesterol acyltransferase-like [*Solanum lycopersicum*]	2/12	8e-27
-4	AAS07227.1	218		putative acyl-activating enzyme [*Oryza sativa* Japonica]	1/12	
-5		216		no significant match	1/12	
ACCT5-1	ACG37370.1	193	−	lysine ketoglutarate reductaselysine trans-splicing [*Zea mays*]	7/12	2e-29
-2	CAA32749.1	193		3-dehydroquinate dehydratase [*Neurospora crassa*]	2/12	4e-05
-3	CAY69152.1	193		AMP-activated serine/threonine protein kinase [*Pichia pastoris* GS115]	2/12	

-4	AAM91233.1	190		glyoxalase II isozyme, putative [*Arabidopsis thaliana*]	1/12	6e-21
ACCT6-1		151	+	no significant match	10/12	
-2		149		no significant match	2/12	
ACCT7-1	BAF62637.1	135	+	DELLA protein [*Phaseolus vulgaris*]	10/12	4e-15
-2		134		no significant match	2/12	
ACCT8-1	AAC78594.1	129	+	Hcr2-2A [*Solanum pimpinellifolium*]	10/12	
-2	CAN66906.1	130		hypothetical protein [*Vitis vinifera*]	2/12	5e-06
ACCT9-1	AAK20059.1	126	+	hypothetical protein [*Oryza sativa* Japonica]	6/10	
-2		127		no significant match	4/10	
ACCT10	AAA74119.1	121	+	SR1 Nt-rab7b [*Nicotiana tabacum*]	12/12	5e-04
ACCT11-1		119	+	no significant match	10/12	
-2	CAN65290.1	119		hypothetical protein [*Vitis vinifera*]	2/12	
ACCT12-1	ABH09088.1	116	−	putative membrane protein [*Artemisia annua*]	6/8	9e-14
-2		117		no significant match	2/8	
ACCT13	AAK11255.1	114	+	regulator of gene silencing [*Nicotiana tabacum*]	10/10	5e-04
ACCT14-1	AAC06242.1	102	−	late embryogenis abundant protein 5 [*Nicotiana tabacum*]	5/12	4e-03
-2	BAD45267.1	102		putative ES2 protein [*Oryza sativa* Japonica]	4/12	
-3	EF588039.1	102		THIC gene[*Nicotiana tabacum*]	3/12	5e-03
ACCT15-1		96	+	no significant match	6/12	
-2	AAA35050.1	97		antiviral protein [*Saccharomyces cerevisiae*]	4/12	
-3		98		no significant match	2/12	
ACGA1-1	ABW98323.1	174	+	hypothetical protein [*Hemiselmis andersenii*]	10/15	
-2	ACG38944.1	173		hypothetical protein [*Zea mays*]	3/15	1e-11
-3	ACG32219.1	171		hypothetical protein [*Zea mays*]	2/15	4e-05

ACGA2-1	CAA77381.1	154	+	ribosomal protein S3 [*Nicotiana tabacum*]	10/17	1e-03
-2	ABU49722.1	153		WRKY transcription factor 3 [*Solanum tuberosum*]	5/17	7e-14
-3	ACG36775.1	156		DNA binding protein [*Zea mays*]	2/17	3e-13
ACGA3-1		151	+	no significant match	6/10	
-2		149		no significant match	4/10	
ACGA4-1	AAL66977.1	141	+	putative cleavage and polyadenylation specificity factor [*Arabidopsis thaliana*]	8/10	
-2		141		no significant match	1/10	
-3		139		no significant match	1/10	
ACGA5	CAN64891.1	135	−	hypothetical protein [*Vitis vinifera*]	7/7	
ACGA6-1		107	−	no significant match	7/14	
-2	AAM20478.1	104		putative protein kinase [*Arabidopsis thaliana*]	4/14	e-11
-3	AAN41309.1	106		unknown protein [*Arabidopsis thaliana*]	3/14	9e-08
ACGA7		97	+	no significant match	8/8	
ACGA8-1	AAF24496.1	93	+	FH protein NFH1 [*Nicotiana tabacum*]	6/8	
-2	CAB10434.1	91		cellulose synthase like protein [*Arabidopsis thaliana*]	2/8	9e-15
ACGA9	AAD25541.	84	+	fructose-1,6-bisphosphatase precursor [*Solanum tuberosum*]	8/8	9e-08
ACGA10-1	BAA25639.1	76	−	NPCA1 [*Nicotiana paniculata*]	8/10	8e-04
-2	ACJ85320.1	77		MTYFD_FE_FF_FG1G-F-5 unknown mRNA.	2/10	5e-04
ACGT1	AAK13103.1	285	−	Helicase-like protein [*Oryza sativa* Japonica]	6/6	2e-03
ACGT2-1	CAA41713.1	193	−	photosystem II 23 kDa polypeptide [*Nicotiana tabacum*]	5/6	3e-20
-2		197		no significant match	1/6	
ACGT3-1	AAA34053.1	190	+	beta-1,3-glucanase [*Nicotiana tabacum*]	5/6	2e-15
-2		191		no significant match	1/6	

ACGT4	AAN63619.1	188	+	thioredoxin h-like protein [*Nicotiana tabacum*]	6/6	4e-09
ACGT5-1	CAB80270.1	183	+	hypothetical protein putative protein [*Arabidopsis thaliana*]	6/15	3e-05
-2	BAE99340.1	184		RNA helicase like protein [*Arabidopsis thaliana*]	4/15	1e-17
-3	CAN77525.1	183		hypothetical protein [*Vitis vinifera*]	2/15	
-4	BAF65022.1	183		hypothetical protein [*Cycas taitungensis*]	2/15	6e-20
-5	AAC67322.1	185		unknown protein [*Arabidopsis thaliana*]	1/15	4e-11
ACGT6		181	−	no significant match	8/8	
ACGT7-1	CAA77408.1	165	+	ribosomal protein L23 [*Nicotiana tabacum*]	6/10	2e-25
-2	AAN41316.1	168		unknown protein [*Arabidopsis thaliana*]	3/10	
-3	AAD03672.1	167		resistance protein candidate RGC2K [*Lactuca sativa*]	1/10	
ACGT8-1	CAJ32461.1	160	+	putative chloroplast cysteine synthase 1 [*Nicotiana tabacum*]	10/15	1e-08
-2	AAK96063.2	160		1-deoxy-D-xylulose-5-phosphate reductoisomerase [*Solanum lycopersicum*]	3/15	5e-22
-3	AAR99489.1	160		PHO1-like protein [*Arabidopsis thaliana*]	1/15	
-4	AAN15455.1	159		putative protein [*Arabidopsis thaliana*]	1/15	
ACGT9-1	CAX42612.1	143	+	NADPH-dependent 1-acyl dihydroxyacetone [*Candida dubliniensis* CD36]	7/10	3e-23
-2	BAC41900.1	143		putative protein kinase [*Arabidopsis thaliana*]	2/10	
-3	BAC20879.1	142		putative IkappaB kinase complex-associated protein [*Oryza sativa* Japonica]	1/10	
ACGT10	CAD30209.1	140	+	putative auxin-induced protein 29 [*Arabidopsis thaliana*]	10/10	
ACGT11-1	CAA69901.1	118	+	plasma membrane polypeptide [*Nicotiana tabacum*]	8/10	1e-05
-2	AAN18139.1	119		At4g20170/F1C12_90 [*Arabidopsis thaliana*]	2/10	1e-06
ACGT12	CAA44267.1	115	−	lipid transferase [*Nicotiana tabacum*]	8/8	6e-10

ACCA1-1	AAD27634.1	253	+	hypothetical protein [*Oryza sativa* Indica]	5/10	
-2	BAA10929.1	252		cytochrome P450 like_TBP [*Nicotiana tabacum*]	3/10	
-3		254		no significant match	1/10	
-4		250		no significant match	1/10	
ACCA2-1	AAN41377.1	204	+	unknown protein [*Arabidopsis thaliana*]	6/10	5e-06
-2	CAN76136.1	204		hypothetical protein [*Vitis vinifera*]	4/10	2e-07
ACCA3	CAA45741.1	200	+	mRNA C-7 [*Nicotian tabacum*]	8/8	6e-11
ACCA4	BAA28625.1	194	+	aldehyde oxidase [*Arabidopsis thaliana*]	10/10	2e-07
ACCA5-1	AAK40224.1	190	+	putative syntaxin of plants 52 [*Oryza sativa* Japonica]	8/10	1e-08
-2	BAC99744.1	190		no significant match	2/10	
ACCA6	AAM65499.1	186	+	AP2 domain transcription factor [*Arabidopsis thaliana*]	8/8	2e-05
ACCA7-1	ABG73415.1	185	+	chloroplast pigment-binding protein CP29 [*Nicotiana tabacum*]	5/10	4e-05
-2	AAG60196.1	185		unknown protein [*Oryza sativa* Japonica]	3/10	
-3	AAO30046.1	185		unknown protein [Arabidopsis thaliana]	2/10	
ACCA8	ABD28323.2	183	+	excinuclease ABC, C subunit[*Medicago truncatula*]	10/10	4e-08
ACCA9-1	AAM91702.1	174	+	unknown protein [*Arabidopsis thaliana*]	4/12	5e-14
-2	ABG73415.1	174		chloroplast pigment-binding protein CP29 [*Nicotiana tabacum*]	3/12	4e-05
-3	CAA77361.1	176		ribulose-1,5-bisphosphate carboxylase/oxygenase [*Nicotiana tabacum*]	1/12	7e-26
-4	BAC79675.1	176		putative translation releasing factor2 [*Oryza sativa* Japonica]	1/12	2e-20
-5	BAC53932.1	175		hypothetical protein [*Nicotiana tabacum*]	1/12	2e-10
-6		175		no significant match	1/12	
-7	BAD15086.1	173		CCAAT-box binding factor HAP2 [*Daucus carota*]	1/12	2e-35
ACCA10	AAM73656.1	168	+	AER [*Nicotiana tabacum*]	6/6	2e-22
ACCA11-1	ABN09771.1	139	−	glycosyl transferase, family 48 [*Medicago truncatula*]	10/16	1e-13

-2		138		no significant match	2/16	
-3	BAF34116.1	138		glycerophosphodiesterase-like protein [*Nicotiana tabacum*]	2/16	8e-05
-4	ABN68186.2	140		G2-specific serine/threonine protein kinase [*Pichia stipitis* CBS 6054]	1/16	2e-13
-5	CAN61131.1	139		hypothetical protein [*Vitis vinifera*]	1/16	1e-05
ACCA12-1		126	+	no significant match	10/14	1e-05
-2	ACG35341.1	128		ATP binding protein [*Zea mays*]	2/14	
-3		126		no significant match	2/14	
ACAG1	CAA74359.1	269	−	ferredoxin--NADP(+) reductase [*Nicotiana tabacum*]	10/10	7e-38
ACAG2-1	CAC81898.1	218	+	NEP1-interacting protein 2 [*Arabidopsis thaliana*]	9/10	5e-07
-2		220		no significant match	1/10	
ACAG3-1		186	+	no significant match	7/12	
-2		185		no significant match	3/12	
-3	BAD83479.1	187		hypothetical protein [*Nicotiana tabacum*]	2/12	9e-14
ACAG4-1	AAD32145.1	176	−	Nt-iaa4.5 deduced protein [*Nicotiana tabacum*]	9/10	3e-05
-2	AAP37824.1	179		At3g54370 [*Arabidopsis thaliana*]	1/10	1e-04
ACAG5-1	AAM28014.1	158	+	granule-bound starch synthase [*Peraphyllum ramosissimum*]	7/16	1e-07
-2	CAN67006.1	158		hypothetical protein [*Vitis vinifera*]	5/16	7e-06
-3	ACG33477.1	157		brassinosteroid-insensitive 1-associated [*Zea mays*]	2/16	4e-11
-4	CAA88339.1	155		nuclear protein SON1 (L00928) [*Saccharomyces cerevisiae*]	2/16	3e-09
ACAG6	BAD46202.1	131	+	hypothetical protein [*Oryza sativa* Japonica]	8/8	
ACAG7-1		115	−	no significant match	5/8	
-2		115		no significant match	2/8	
-3		114		no significant match	1/8	
ACAG8	AAY17071.1	108	−	chloroplast carbonic anhydrase [*Nicotiana benthamiana*]	6/6	5e-21

ACAG9	CAA44267.1	97	−	lipid transferase [*Nicotiana tabacum*]	10/10	7e-08
ACAG10	AAA34065.1	79	−	chloroplast carbonic anhydrase [*Nicotiana tabacum*]	10/10	1e-07
ACAG11-1	BAC79914.1	57	−	homeobox transcription factor Hox7-like protein [*Oryza sativa* Japonica]	5/10	
-2		54		no significant match	3/10	
-3		55		no significant match	2/10	
ACAG12-1		47	+	no significant match	6/10	
-2		48		no significant match	4/10	

[a]TDF: transcript-derived fragment

[b]ID: accession number of Tblastx homologue

[c]Expression: up-regulated (+) or down-regulated (-) cDNA-AFLP signals detected in virus-infected leaves, compared to mock-infected leaves

[d]Protein candidate: Tblastx hit with the best E value

[e]Ratio: the number of the clone over the number of total clones sequenced

[f]E value: only the value lower than 0.001 (1e-03) were shown according to statistic analysis with extremely significant hit

Table S2. The primer set and their sequence for RT-PCR to examine the knockdown efficiency.

primer	sequence
**ACCT13F**<br>**ACCT13R**	**5'GAAAGAATGAGCAATCCTCAA3'**<br>**5'GATATTTGTTAATTTGATGGCAGAA3'**
**ACGT4F**<br>**ACGT4R**	**5'GACATAAAAGCTACTCCAACCTT3'**<br>**5'GCTTAATATGCATAGAAACACGT3'**
**ACGT11-1F**<br>**ACGT11-1R**	**5'GACTAAGTCTCCCACCTAAAC3'**<br>**5'GATCAATTGGTGCTTACCACCTTC3'**
**ACAG2-1F**<br>**ACAG2-1R**	**5'CTAACATTTTCGATACAG3'**<br>**5'CTCAACAGCCACGTATCAATGC3'**
**ACCA10F**<br>**ACCA10R**	**5'GGATCCATGCCTTCTTCTTCAGTTTTTG3'**<br>**5'GTTAGGTACCAGAAACATATTGCATAAATTC3'**
**ACCT1-1F**<br>**ACCT1-1R**	**5'GCGCCTACTCTAGTTCTAGAT3'**<br>**5'GGTAACTATTGGCACCATATATT3'**
**ACCT5-1F**<br>**ACCT5-1R**	**5'GGTGAACCAAGTGAGGATCTG3'**<br>**5'GAAAGAAGCAATGTACAGAACA3'**
**ACAG8F**<br>**ACAG8R**	**5'GAAGGAAGCTGTGAATGTGTCA3'**<br>**5'TGGTTAAGTTCATACGGAAAGA3'**
**ACGT2-1F**<br>**ACGT2-1R**	**5'GAGTTTAGAATTATGTCAAAGAGA3'**<br>**5'CTCCAAATATATAGTAGCAGCTA3'**
**ACAG1F**<br>**ACAG1R**	**5'GAGAAAATGAAGGAGAAGGCCC3'**<br>**5'GCTCTGCCTTCTTCAATTGCTTCTT3'**
**ACGT12F**<br>**ACGT12R**	**5'GGATCCATGGAAATAGCTGGGAAAATTG3'**<br>**5'GTTACTCGAGCTGGACCTTGGAGCAGTC3'**
**ACTC5-1F**<br>**ACTC5-1R**	**5'GTTAGAGTAGCCAAAGCCATTGGAA3'**<br>**5'GTGCAAGTATTTTACGGTTAAGAAGC3'**
**ACCT3-1F**<br>**ACCT3-1R**	**5'GCGCCAATGTTTTTCAAGGGA3'**<br>**5'GAACTGTTTCAAAGACCCAA3'**
**ACCA3F**<br>**ACCA3R**	**5'GGATCCATGGCTGACGAAGTTGTCC3'**<br>**5'GTTACTCGAGGGCAATGCCCCACTTTTG3'**
**ACGA3-1F**<br>**ACGA3-1R**	**5'GCTTGTGGAGATGCTTTGTGT3'**<br>**5'CATGAACAATCTCCCATTATAATTT3'**
