# Supplementary figures and images for: Identification of differentially expressed genes induced by Bamboo mosaic virus infection in Nicotiana benthamiana by cDNA-amplified fragment length polymorphism
Source: BMC Plant Biol. 2010 Dec 27;10:286. doi: 10.1186/1471-2229-10-286 (PMC3024324; doi:10.1186/1471-2229-10-286)

Figure S1

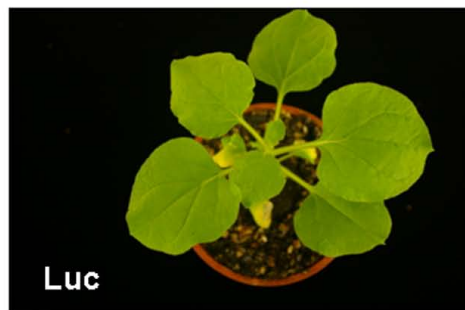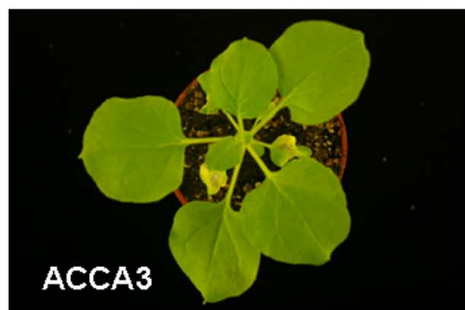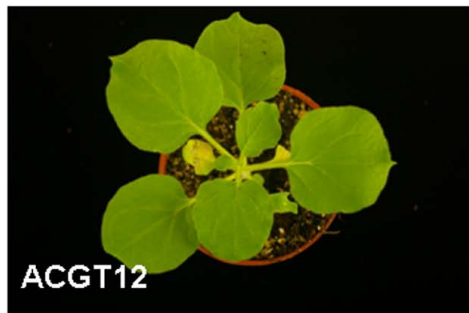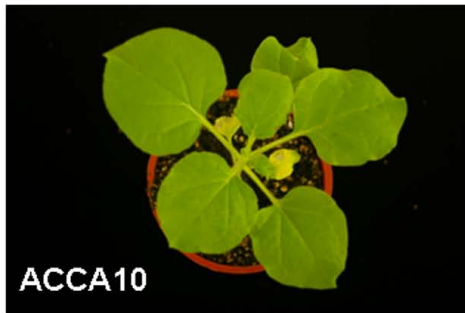

Figure S2

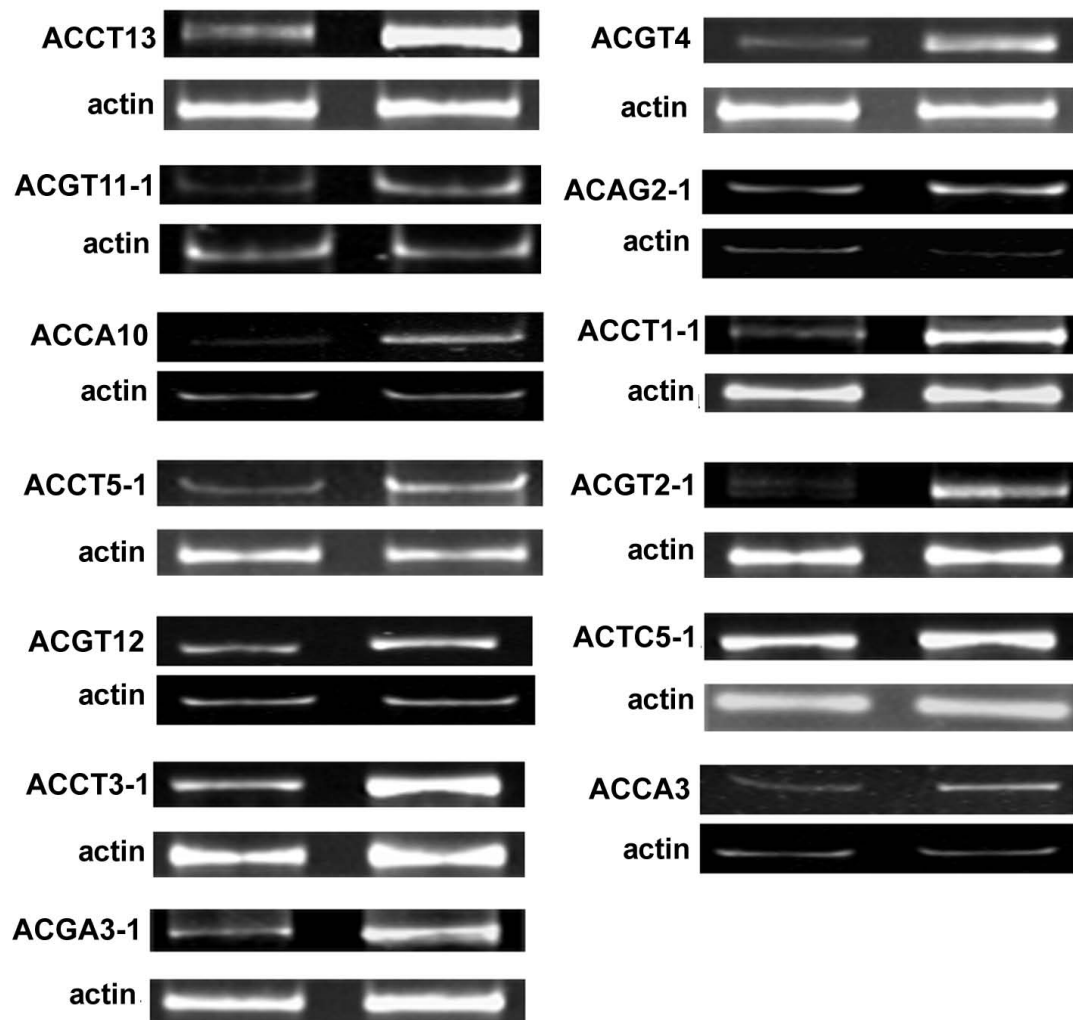

Supplement: Additional file 2 — Figure S1 - Phenotypes of gene-specific knockdown plants generated by the TRV VIGS system. Transcription of ACCA3, ACGT12, or ACAG8 in N. benthamiana plant was introduced by the TRV vector to knock down expression of the corresponding host genes. The Luc plant in which the luciferase gene was introduced was included as a negative control. Figure S2 - RT-PCR analysis of host gene expression in knockdown plants. The knockdown plants as indicated above each lane were inoculated with viral RNA. The GFP plant was included as the negative control. The RNA extracts derived from the leaves inoculated with viral RNA were harvested on 5 dpi and subjected to specific primers indicated on the left for semi-quantitative RT-PCR. RT-PCR data was normalized to the levels of actin. [file 1471-2229-10-286-S2.PDF]
